# Transylvania Medical School

**Published:** 1844-05

**Authors:** 


TRANSYLVANIA MEDICAL SCHOOL.

This institution has lately been the subject of an animated controversy in one of the Lexington papers. A graduate of the school, an experienced and highly intelligent physician of Lexington, calls upon the Trustees to remedy certain organic defects in the institution which, he contends, seriously threaten its prosperity, if not its very existence. He says:

"This school, (Transylvania) which is the oldest in the West, had no rival for many years, during which time it acquired a high reputation, but from the operation of causes which are now well known, it has commenced with fearful rapidity a decline in public estimation, and in the estimation even of its friends, which will soon ultimate in its entire dissolution and abandonment, unless, by timely re-organization, the causes producing this effect are counteracted or removed.

"The writer of this communication is a graduate of Transyvania, and not only regrets the indications of her declining reputation, but desires the prosperity, the distinguished and unfading renown of his parent institution; and the object he has in view in writing this article is to call the attention of the trustees of Transylvania, and citizens of Lexington, to the fact of its decline, that they, who are her guardians and who have power to act efficiently for her welfare, may speedily do it, before it be too late, by removing the causes which are hastening her dissolution.

"By comparing the last catalogue of this school with those of Cincinnati and Louisville, it will be perceived that the former is lecturing to nearly as many students, if not quite, as Transylvania, and that the latter has greatly the ascendency. Why is it that the Medical Institute of Louisville numbers 242 students, and this not quite 200?"

He enters, then, into the discussion of the causes which have brought about this result, and adds:

"When the medical department of Transylvania University was organized, a capital error was committed in blending the professorships of anatomy and surgery, and appointing the same individual to both professorships, and requiring him to do what no man is competent to do within the short period of four months, except imperfectly, to teach both anatomy and surgery.

"In the Louisville Institute a gentleman of distinguished talents and acquirements devotes the whole of his time to anatomical lectures, and such is the just appreciation of the value and importance of thorough knowledge upon this subject, he is assisted by a demonstrator of anatomy of equal talents with himself.

"This being the fact in relation to the facilities of obtaining a full knowledge of anatomy in the Louisville Institute, I ask, do not the friends of Transylvania see that unless equal facilities and advantages are afforded by our school, it must continue to fall, and the other rise, until there will be no competition between them.

"In the Louisville school, a gentleman of learning, talents, and of acknowledged practical abilities as a lecturer and operator, devotes the whole of his time to teaching the important branch of surgery, and in Transylvania the professor of anatomy and surgery divides his time between both subjects, and cannot, for want of time, if nothing else were wanting, teach either branch, if he had the talents of both, more than half so extensively and thoroughly as they are taught in our rival institution.

"We have conversed with individuals who attended both schools, who were competent to judge between them, who affirm that more than double the amount of anatomy and surgery are taught at the Louisville Institute, and as well taught, not to say better, than in Transylvania; and, with a knowledge of these facts, young gentlemen, in pursuit of medical knowledge, have gone there in preference to coming to Lexington, until it has become our successful rival, and unless by a speedy *re-organization*, which will afford at least equal facilities, and an equal amount of anatomical and surgical knowledge in our school, it will soon cease to be even the second institution in the West, instead of being the first. That a professor of anatomy of acknoledged abilities, who shall devote the whole of his time to that branch, is needed in Transylvania is evident from the dissatisfaction which the students generally expressed within the last session, many of whom were known to say they had visited this institution for the first and last time, unless there was a new arrangement by which they could obtain as much anatomical and surgical instruction here as at Louisville."

Dr. Peter deemed these complaints of so grave a nature as to require an answer from him, and he is accordingly out, at much length, in the same paper. He denies that the school has fallen into "a rapid decline," and publishes statistics to show that it has not lost quite sixty students in two years. He insists, in reply to his neighbor, that "Dr. Dudley, without descending into the trifling *minutiæ* of *unimportant* surgical and anatomical *niceties*, gives the result of his unequalled practical experience in the most simple and impressive manner." He considers it possible that "other surgeons may appear more learned in authors and opinions, or on the number of tails of a bandage;" but only let it come to cutting "the gordian knot of conflicting authorities," and, he assures us, "Dr. Dudley has no rival." He concludes his defence by asking his antagonist a question which, it is quite plain, he considers unanswerable, and by which, doubtless, he expected to terminate the controversy.

"If," says he "Dr. Dudley cannot teach anatomy and surgery, *who can?*"

Now, *non nostrum tantas componere lites.* We have no wish to interfere in the strifes of our neighbors, out of which we have little expectation of seeing anything grow of benefit to that school, or to the profession. We should not have felt authorized to refer to this controversy at all, if Dr. Peter, quitting the defence of Dr. Dudley, had not taken occasion to allude, in very offensive terms, to some of the professors in the Medical Institute of Louisville. We hope he was betrayed into the indecorum by hasty feeling, and that in his subsequent cool reflections he has found cause to deplore it. He must be satisfied that the late embittered strife between the rival schools was kept up quite too long, and that no good can result from its revival. He must have been aware that his language in reference to members of the Medical Institute, is well calculated to lead to a renewal of personal quarrels of which the public is tired, and by which neither party is likely to gain anything. Peace, assuredly, is the policy of both institutions, as it is the only line of dignity. Let the members of each labor faithfully in their places to build up schools which shall be ornaments and blessings to our country.

We do not doubt, that we shall have the feelings of all right thinking men with us upon this subject. They will condemn such an aggression by a professor in one school upon the members of a rival institution. It is the desire of the profession to see harmony among our numerous schools of medicine, and we are sure they will disapprove of any act which tends to disturb it. Y.

